# Selectivity and self-diffusion of CO_2_ and H_2_ in a mixture on a graphite surface

**DOI:** 10.3389/fchem.2013.00038

**Published:** 2013-12-24

**Authors:** Thuat T. Trinh, Thijs J. H. Vlugt, May-Britt Hägg, Dick Bedeaux, Signe Kjelstrup

**Affiliations:** ^1^Department of Chemistry, Norwegian University of Science and TechnologyTrondheim, Norway; ^2^Department of Process and Energy, Delft University of TechnologyDelft, Netherlands; ^3^Department of Chemical Engineering, Norwegian University of Science and TechnologyTrondheim, Norway

**Keywords:** CO_2_−H_2_ mixture, adsorption, diffusion, molecular dynamics simulation, graphite

## Abstract

We performed classical molecular dynamics (MD) simulations to understand the mechanism of adsorption from a gas mixture of CO_2_ and H_2_ (mole fraction of CO_2_ = 0.30) and diffusion along a graphite surface, with the aim to help enrich industrial off-gases in CO_2_, separating out H_2_. The temperature of the system in the simulation covered typical industrial conditions for off-gas treatment (250–550 K). The interaction energy of single molecules CO_2_ or H_2_ on graphite surface was calculated with classical force fields (FFs) and with Density Functional Theory (DFT). The results were in good agreement. The binding energy of CO_2_ on graphite surface is three times larger than that of H_2_. At lower temperatures, the selectivity of CO_2_ over H_2_ is five times larger than at higher temperatures. The position of the dividing surface was used to explain how the adsorption varies with pore size. In the temperature range studied, the self-diffusion coefficient of CO_2_ is always smaller than of H_2_. The temperature variation of the selectivities and the self-diffusion coefficient imply that the carbon molecular sieve membrane can be used for gas enrichment of CO_2_.

## Introduction

The production of cheap membranes for CO_2_ gas separation purposes is of primary importance for the realization of carbon capture and sequestration technologies (He et al., 2009; He and Hägg, 2011, 2012). One of the important applications of membranes is to separate CO_2_ from a mixture of gases (Bernardo et al., 2009). Pressure swing adsorption (PSA) is one of the most common techniques to capture CO_2_ from a mixture of CO_2_ and H_2_. This process requires large pressures, being different in the adsorption and desorption steps (Bernardo et al., 2009). In the adsorption step, CO_2_ absorbs strongly into the carbon material at high pressure. Then in the later step, CO_2_ desorbs at a much lower pressure. The energy costs depend on the manner this is performed; in one or more steps, with or without heat integrated. By using a molecular sieve membrane the separation can be performed as a continuous process, where the CO_2_ is removed both by adsorption and diffusion from the high pressure side (feed side) to the low pressure side (permeate side). To provide an energy efficient design, we will need knowledge of molecular behavior, in particular of the selectivity and of transport properties at selected process conditions. Although there is a lot of recent progress in the modification of graphene material for adsorption and separation application of CO_2_ and H_2_ these material are not cheap (Kim et al., 2013; Li et al., 2013). Nano-porous, fibrous, carbonaceous materials are promising candidates from an economic point of view. In order to make further progress and produce molecular sieve membranes, better knowledge of several issues is needed. Central for membrane functionality are pore size, surface binding, surface wall transport, pore inlet control, carbon structure and composition. This work aims to provide such knowledge for a simplified, graphitic membrane, laying the grounds for more realistic future studies.

There are several experimental works and simulations devoted to understand adsorption of single component CO_2_ and H_2_ on carbon based material such as activated carbon and graphite (Guo et al., 2006; Haas et al., 2009; Levesque and Lamari, 2009; Jin et al., 2011; Saha et al., 2011). The experimentally obtained adsorption isotherm of CO_2_ on active carbon is well-described by several models such as Langmuir (Jin et al., 2011), Tóth (Himeno et al., 2005), Dubinin-Astakhov (D-A) (Saha et al., 2011; Sevilla and Fuertes, 2012). Reported values for the enthalpy of adsorption depend on the type of adsorbent. Saha et al. reported that heats of adsorption of CO_2_ in Maxsorb II and ACF (A-20) material are around −20 kJ/mol (Saha et al., 2011), while the untreated activated carbon C3345 material has a heat of adsorption −14 kJ/mol (Jin et al., 2011). Guo et al. reported that the heat of adsorption varied in the range −10 to −28 kJ/mol depending on the modification of the activated carbon material (Guo et al., 2006). Himeno et al. reported adsorption enthalpies in the range −16 to −25 kJ/mol for pure CO_2_ on five different commercial activated carbons (Himeno et al., 2005).

Several simulation studies have given the adsorption isotherms for CO_2_ on planar and pore-like graphite surfaces. Lim et al. (2010) reported data using a Langmuir adsorption model, and provided the self-diffusion coefficient (*D*_*s*_= 10^−8^−10^−9^ m^2^/s) of CO_2_ in a narrow pore (width 0.65–0.75 nm) for temperatures *T* = 298−318 K. Zhou et al. reported results for a wider range of pore sizes (0.7–3.4 nm) (Zhou and Wang, 2000). Their values are comparable with those of Lim et al. The authors reported that CO_2_ could form double layers. Both layers had a typical liquid-like structure (Zhou and Wang, 2000). Levesque et al. calculated the heat of CO_2_ adsorption on activated carbon using Monte-Carlo simulations (Levesque and Lamari, 2009). The authors discussed how the adsorption enthalpy depended on the distribution of pore sizes.

Adsorption and diffusion of single component H_2_ on graphite have recently been measured (Haas et al., 2009; Simon et al., 2010). The self-diffusion coefficient of H_2_ on a graphite surface was found, using quasielastic neutron scattering (QENS) (Haas et al., 2009), to be in the range 10^−6^−10^−7^ m^2^/s. Simulations found that pure H_2_ on the graphite surface had a high lateral mobility (Simon et al., 2010).

Few computational results are reported on the selective adsorption of a mixture of CO_2_ and H_2_ on a graphite surface. Cao et al. described the graphite surface selectivity of the mixture at bulk compositions 50:50 and 20:80 at three different temperatures, slit pore sizes up to 3.0 nm and pressures up to 10 atm, using Monte Carlo simulations (Cao and Wu, 2005). The selectivity of CO_2_ over H_2_ depended on the pore size and the temperature. More recently, Kumar et al. (Vasanth Kumar and Rodríguez-Reinoso, 2012) reported results for CO_2_/H_2_ mixtures for molar ratios 10:90 and 20:80 on different graphite structures (nanotube, slit pores, or computer generated) at room temperature 298 K. It was shown that mixture separation was best with nanotubes. There are few studies on diffusion of CO_2_ and H_2_ on the graphite surface.

**Figure 1:**
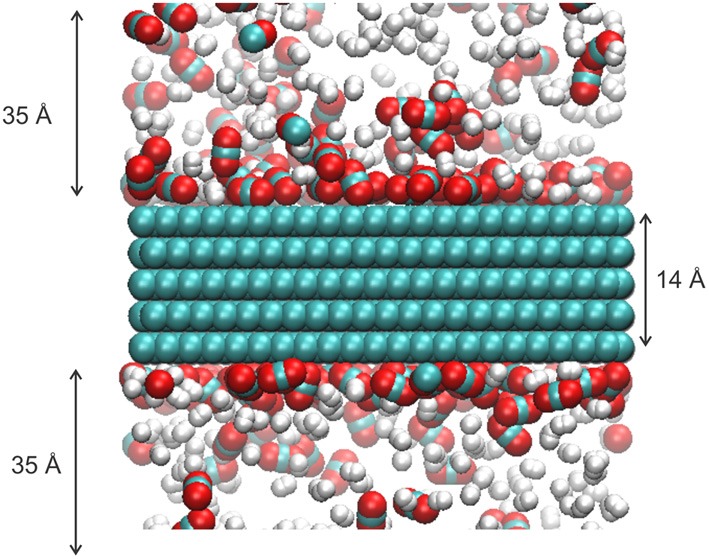
**Typical snapshot of the gas mixture of CO_2_ and H_2_ in equilibrium with a graphite surface**. The temperature is *T* = 300 K and the number of particles is *N* = 700. The green, red, white are represented carbon, oxygen, and hydrogen atom, respectively.

These studies give a motivation for the present work. We want to add to the knowledge of adsorption isotherms for a mixture CO_2_ and H_2_ at a typical syngas compositions (Rostrup-Nielsen and Christiansen, 2011) (mole fraction of CO_2_ = 0.30) on a graphite surface, find the selectivity and self-diffusion coefficient for the components along the surface, and study these properties for a wide range of temperatures (*T* = 250, 550 K). Molecular dynamics (MD) simulations are well-suited to determine such properties. A snapshot of the gas mixture in equilibrium with the graphite is shown in Figure 1.

## Model

### Isotherm adsorption

The reaction between the gas phase and the adsorbed phase on the surface can be written for each component:

(1) $${\text{CO}}_{2}(\text{gas})\text{ + graphite}⇌{\text{CO}}_{2}(\text{graphite})$$

(2) $${\text{H}}_{2}(\text{gas})\text{ + graphite}⇌{\text{H}}_{2}(\text{graphite})$$

At equilibrium, the ideal gas chemical potential is equal to the surface chemical potential for each component:

(3) $$\mu_{g}=\mu_{s}$$

(4) $$\mu_{g}=\mu_{g}^{0}+RT\ln\left(\frac{p}{p_{0}}\right)$$

where μ^0^_*g*_ is the standard chemical potential of the gas phase, i.e., the ideal chemical potential at the reference pressure *p*_0_.

The chemical potential for the surface is

(5) $$\mu_{s}=\mu_{s}^{*}+RT\ln\left(\gamma \frac{\Gamma }{\Gamma^{*}}\right)$$

where μ^*^_s_ is the standard state chemical potential, γ is the activity coefficient, and Γ and Γ ^*^ are the surface adsorption and standard surface adsorption, respectively. The surface adsorption is an excess quantity (see Equation 14 below for the definition).

### Simulation details

To model the CO_2_ adsorption and transport on the graphite surface, we performed classical MD simulations using the DL_POLY classic version 2.18 package (Smith et al., 2002). The system consisted of a graphite crystal and a mixture of CO_2_ and H_2_ molecules, ratio 30:70, an example of a syngas mixture (Rostrup-Nielsen and Christiansen, 2011). The graphite had hexagonal structure with P63/mmc without any defects. The graphite contained 4284 carbon atoms and was constructed from 5 sheets of graphene which represented the property of graphite well (Boukhvalov et al., 2008). We oriented the graphene sheets in our simulation box such that the surfaces of the sheets were perpendicular to the *z* direction. The size of the simulation box was 42 × 51 × 84 Å^3^. In the *z* direction, the system covers a pore size of 70 Å and a graphite width of 14 Å (Figure 1). Periodic boundary conditions are used in all directions. At least 10 systems with different total number (*N*) of molecules, where 10 < *N* < 7.0 were simulated. For each *N*, simulations were performed at 8 different temperatures in the range 250–550 K.

The MD simulation had time steps of 1fs. The initial configuration was constructed by randomly distributing the CO_2_/H_2_ mixture over the graphite surface. The system was stabilized during 1000 ps by *NVT* runs with the Nosé-Hoover thermostat (Martyna et al., 1992). When the system was in the thermal equilibrium, we performed another 1000 ps run with microcanonical ensemble conditions (*NVE*) to study adsorption and transport properties. The average values of temperature and pressure in *NVE* simulation were within 1% of expected values. In total 2 × 10^6^ MD steps was performed and this is sufficiently long to get good statistics and consistent trajectories. Each trajectory was printed every 100 time step and stored for further analysis.

### Potential energy interaction

We fixed the graphite layer and used the rigid body model of Transferable Potentials for Phase Equilibria (TraPPE) for CO_2_ and H_2_ molecule. This potential can describe well the vapor-liquid and the liquid-solid equilibria of CO_2_ (Potoff and Siepmann, 2001). The intermolecular potential contained long range Coulombic interactions, and a shifted and truncated 12-6 Lennard-Jones (LJ) potential (Allen and Tildesley, 1989).

(6) $$V_{ij}^{nb}=V_{ij}^{\text{LJ}}+V_{ij}^{\text{coulombic}}$$

(7) $$V_{ij}(r_{ij})=4\varepsilon_{ij}\left[{\left(\frac{\sigma_{ij}}{r_{ij}}\right)}^{12}-{\left(\frac{\sigma_{ij}}{r_{ij}}\right)}^{6}\right]$$

(8) $$V_{ij}^{\text{LJ}}(r_{ij})=\{\begin{array}{ll} V_{ij}(r_{ij})-V_{ij}(r_{c}) & r_{ij}<r_{c}\\ \;0 & r_{ij}>r_{c} \end{array}$$

where *r*_*ij*_ is the distance between atoms *i* and *j*, ε_*ij*_, and σ_*ij*_ are LJ potential parameters, and *r*_*c*_ is the cutoff radius. The LJ interaction parameters between the different types of atoms were calculated from the Lorentz-Berthlot mixing rules (Allen and Tildesley, 1989)

(9) $$\varepsilon_{ij}=\sqrt{\varepsilon_{ii}\varepsilon_{jj}}$$

(10) $$\sigma_{ij}=\frac{1}{2}\left(\sigma_{ii}+\sigma_{jj}\right)$$

The Coulombic interactions were:

(11) $$V_{ij}^{\text{coulombic}}=\frac{1}{4\pi \varepsilon_{0}}\frac{q_{i}q_{j}}{r_{ij}}$$

where *q*_*i*_, *q*_*j*_ are the charges on atoms *i*, *j*, and ε_0_ is the dielectric constant. In our work, we use the Smoothed Particle Mesh Ewald technique implemented in the DL_POLY package for the electrostatic interactions, see Essmann et al. (1995) for more details. The parameters, taken from the DREIDING (Mayo et al., 1990) and TraPPE (Potoff and Siepmann, 2001) force fields (FFs), are listed in Table 1.

**Table 1:** **Interaction potential parameters used in simulation**.

**Atom**	**σ (Å)**	**ε /*k*_*B*_ (K)**	**charge (*e*)**
C (in CO_2_)	2.80	27	0.7
O (in CO_2_)	3.05	79	–0.35
C (graphite)	3.34	26	0
H (in H_2_)	2.64	28	0

### Density functional theory (DFT) calculations

To evaluate the results using classical FFs, we also performed DFT to calculate the binding energy of CO_2_ and H_2_ on graphite surface. For the *ab-initio* simulations, DFT optimization and single energy were performed using Quickstep (Vandevondele et al., 2005) which is part of the CP2K program package (http://cp2k.berlios.de, 2011). Quickstep employs the Gaussian and plane waves (GPW) method (Lippert et al., 1997) which makes efficient and accurate density-functional calculations of large systems possible. We used the Goedecker-Teter-Hutter (GTH) pseudopotentials (Goedecker et al., 1996; Hartwigsen et al., 1998) to describe atomic cores and the PBE exchange-correlation functional (Perdew et al., 1996). One-electron wave functions were developed under the DZVP-MOLOPT (DZPM) basis set, offering a double-zeta valence complemented with polarization functions (Vandevondele and Hutter, 2007). An energy cut-off of 400 Ry was selected for the additional plane wave basis sets. To describe the van der Waals interactions, an empirical dispersion correction of Grimme's type was applied (Grimme, 2006).

DFT is a computationally expensive method for a large system. Hence we used a much smaller model than with the FF method. Five sheets of 32 carbon atom each was used to construct the graphite surface. The graphite geometry was chosen similarly to the FF simulation. The system was fully optimized, and then CO_2_ and H_2_ molecules were fixed at selected distance from the surface for single point energy calculations.

We used the DFT method to calculate the interaction energy between each component (CO_2_, H_2_) and graphite surface.

(12) $$E_{{\text{CO}}_{2}}^{i}=E_{{\text{Graphite-CO}}_{2}}-(E_{\text{Graphite}}+E_{{\text{CO}}_{2}})$$

(13) $$E_{{\text{H}}_{2}}^{i}=E_{{\text{Graphite-H}}_{2}}-(E_{\text{Graphite}}+E_{{\text{H}}_{2}})$$

For X = CO_2_ or H_2_:*E*^*i*^_*X*_, *E*_Graphite−*X*_, *E*_Graphite_, *E*_*X*_ are the interaction energy, potential energy of graphite-X system, potential energy of graphite and potential energy of X, respectively.

The optimum distance of adsorption is the distance between molecule and graphite surface where the interaction energy profile has a minimum.

## Results and discussion

### Interactions between CO_2_/H_2_ and the graphite surface

**Figure 2:**
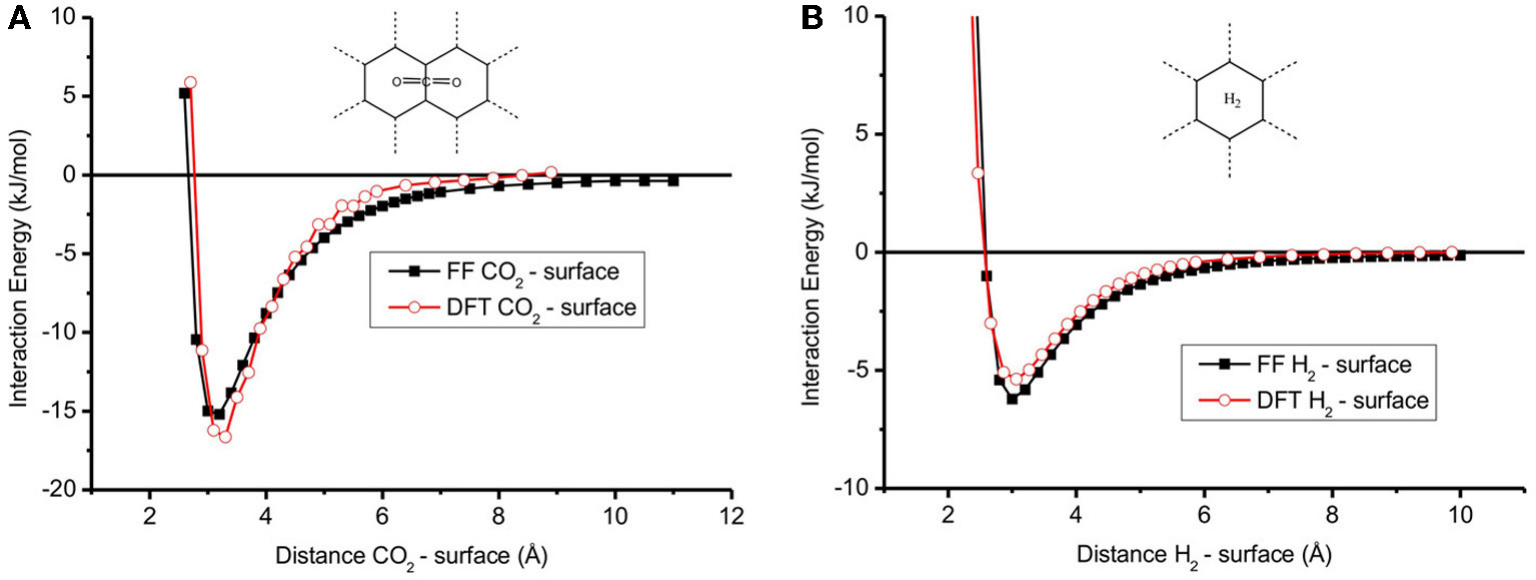
**Interaction energy between (A) CO_2_—graphite surface and (B) H_2_—graphite surface calculated with DFT and Force Field method**. The result of FF method is comparable with DFT method.

Figure 2 shows the adsorption energy profile of a single CO_2_ or H_2_ molecule on the graphite surface calculated with the FF and DFT methods for the optimum molecule-surface distance. This distance and the adsorption energy are given in Table 2 for both methods. CO_2_ is favorably adsorbed at the bridge site and is parallel to the surface, while H_2_ prefers the hollow site and is perpendicular to the surface. Our finding is supported by Rubes et al. (2010). The profiles of the plots in Figure 2 are very similar, meaning that the FF results can be seen as a good representation of the DFT calculations. The values of the interaction energy of CO_2_ and H_2_ on the surface (Table 2) are typical for physisorption. For CO_2_ on surface, the interaction energy *E*^*i*^_CO_2__ = −15 and −17 kJ/mol for FF and DFT, respectively. The optimum distance of CO_2_ and surface is around 3.20–3.30 Å (Table 2). The interaction energy in the case of H_2_ on the surface is smaller than in the case of CO_2_ on the surface (*E*^*i*^_H_2__ = −6 and −5 kJ/mol for FF and DFT, compare Figures 2B to 2A) while the optimum distance of H_2_ and the surface is around 3.0 Å (Figure 2B). Furthermore, the interaction energies are in good agreement with other DFT calculations (Montoya et al., 2003; Rubes et al., 2010), meaning that our results for binding energies and distances are robust.

**Table 2:** **Optimum distance for adsorption and corresponding interaction energies for the FF and DFT methods used**.

**Method**	**Force field**	**DFT PBE-D, periodic**
Optimum distance CO_2_ – surface	3.20 Å	3.30 Å
Interaction energy CO_2_ – surface *E*^*i*^_CO__2_	−15 kJ/mol	−17 kJ/mol
Optimum distance H_2_ – surface	3.0 Å	3.0 Å
Interaction energy H_2_ – surface *E*^*i*^_H__2_	−6 kJ/mol	−5 kJ/mol

### The structure of mixture on surface

**Figure 3:**
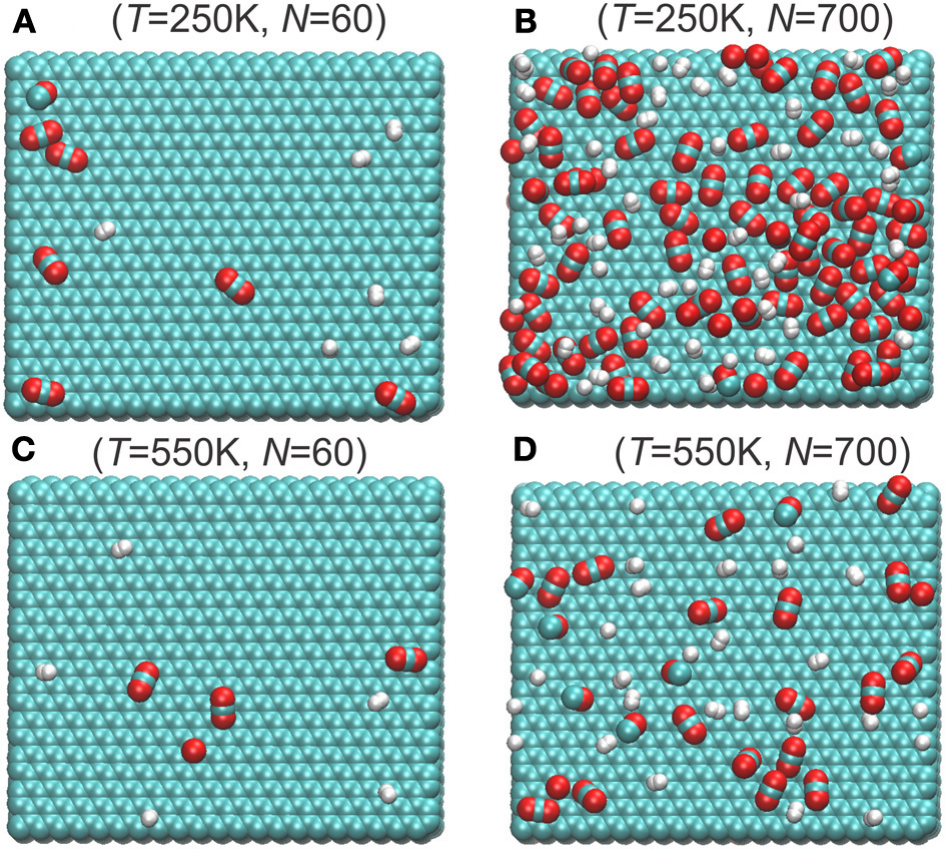
**Typical snaphosts of top view of molecules adsorbed on the graphite surface at different temperatures and total number of particles (A) *T* = 250 K, *N* = 60; (B) *T* = 250 K, *N* = 700; (C) *T* = 550 K, *N* = 60; (D) *T* = 550 K, *N* = 700, cf. Figure 6**. Only the adsorbed layers below the dividing surface are shown (cf. Equation 14). The green, red, white represent carbon, oxygen, and hydrogen atoms, respectively.

Typical snapshots of mixtures adsorbed on graphite surfaces at different temperatures and total number of particles are depicted in Figure 3. With the same number of total molecules, there are more molecules adsorbed on the surface at lower temperature (compare Figures 3A,B) than at higher temperatures (Compare Figures 3C,D). The ratio CO_2_/H_2_ on the graphite surface is also larger in the low temperature range than at high temperatures. H_2_ and CO_2_ appears randomly distributed on the surface when surface has low loading (Figures 3A–C). But when the surface has high loading and CO_2_ is preferred at the surface, the H_2_ molecules seem to occupy the voids between the CO_2_ molecules (Figure 3D).

### Surface excess densities

**Figure 4:**
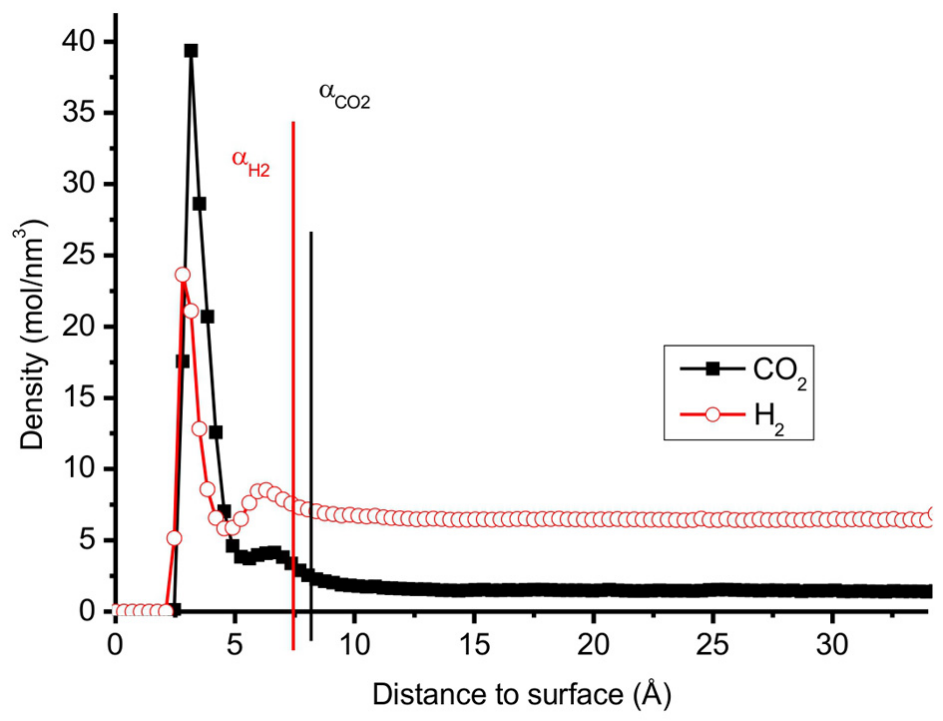
**The distribution of pure CO_2_ and pure H_2_ molecules perpendicular to the surface in a mixture with *N* = 700 at temperature *T* = 300 K**. One can distinguish two regions for each molecule; 0-α: adsorbed layer, above α: gas phase.

In the thermodynamic description, we use the surface excess concentration (adsorption) Γ, as defined originally by Gibbs, see Kjelstrup and Bedeaux (2008) for a detailed description. The interface is defined as the thin layer between phases where densities deviate from the densities in the adjacent phases. We restrict ourselves to surfaces parallel to the graphite surface, so

(14) $$\Gamma =\int_{0}^{\infty }\left(C(z)-C^{\text{gas}}(\alpha )\theta (z-\alpha )\right)dz$$

where Γ is the adsorption, and *C, C*^*gas*^ are the total concentration of molecules and the concentration in the gas, respectively (Figure 4). The Heaviside function, θ, is by definition unity, when the argument is positive, and zero when argument is negative. The extension of the surface can differ from molecule to molecule, as illustrated for the two molecules in question in Figure 4, and a choice must be made. The positions α_H__2_ and α_CO__2_ are defined as the positions where the concentrations of H_2_ and CO_2_ are 5% above the bulk value. For the CO_2_/H_2_ mixture, we choose the dividing surface α = α_CO_2__ as given in the figure for the integral in Equation (14). Adsorption isotherms (Figures 6, 7 below) were obtained by plotting the surface excess concentration provided by Equation (14) for both components using this position, vs. the gas pressure. The gas pressures of CO_2_ and H_2_ were obtained by separate calculations where the simulation box contained only CO_2_ or H_2_ at different temperatures and concentrations. The total gas pressure is the sum of partial pressure of CO_2_ and H_2_.

**Figure 5:**
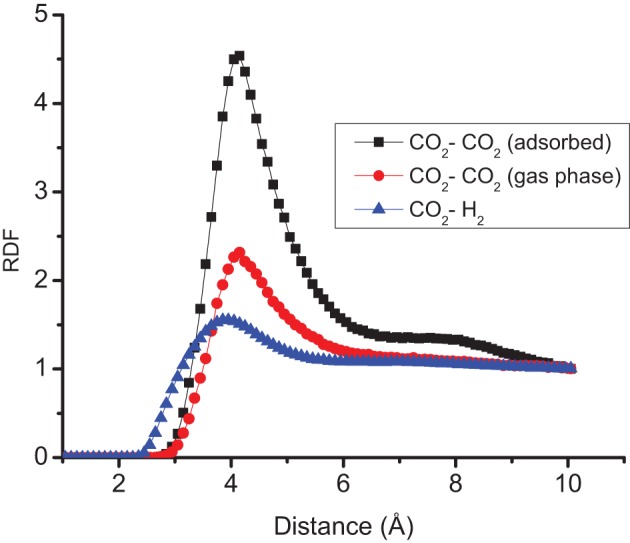
**Radial distribution function RDF of CO_2_–H_2_, CO_2_–CO_2_ in the adsorbed layer and CO_2_–CO_2_ in the gas phase at system *T* = 300 K, *N* = 700, and ^*x*^ CO_2_ = 0.30**.

The distributions of CO_2_ and H_2_ molecules perpendicular to the surface, have two peaks, see Figure 4. The first peak of CO_2_ is located around 3.2 Å and the first of H_2_ is located around 3.0 Å. These peaks correspond to the optimum distances of adsorption of the gas molecules as described in the previous section. The radial distribution functions (RDF) of CO_2_–H_2_ and CO_2_–CO_2_ molecules of the different layers across the surface are reported in Figure 5. The RDF of CO_2_–CO_2_ in the adsorbed phase has a liquid-like behavior and this agrees with other simulations of pure CO_2_ on graphite surface (Zhou and Wang, 2000). The RDF of CO_2_–CO_2_ in the gas phase is less ordered, showing a homogenous behavior. The positions of maximum RDF of the adsorbed layers and the gas of CO_2_ are comparable. The RDF of CO_2_–H_2_ has a typical gas-like behavior, which indicates that CO_2_–H_2_ is near an ideal mixture. The interactions between the gas components are not as important as the interactions between the gas and the graphite surface.

### The adsorption on a graphite surface

**Figure 6:**
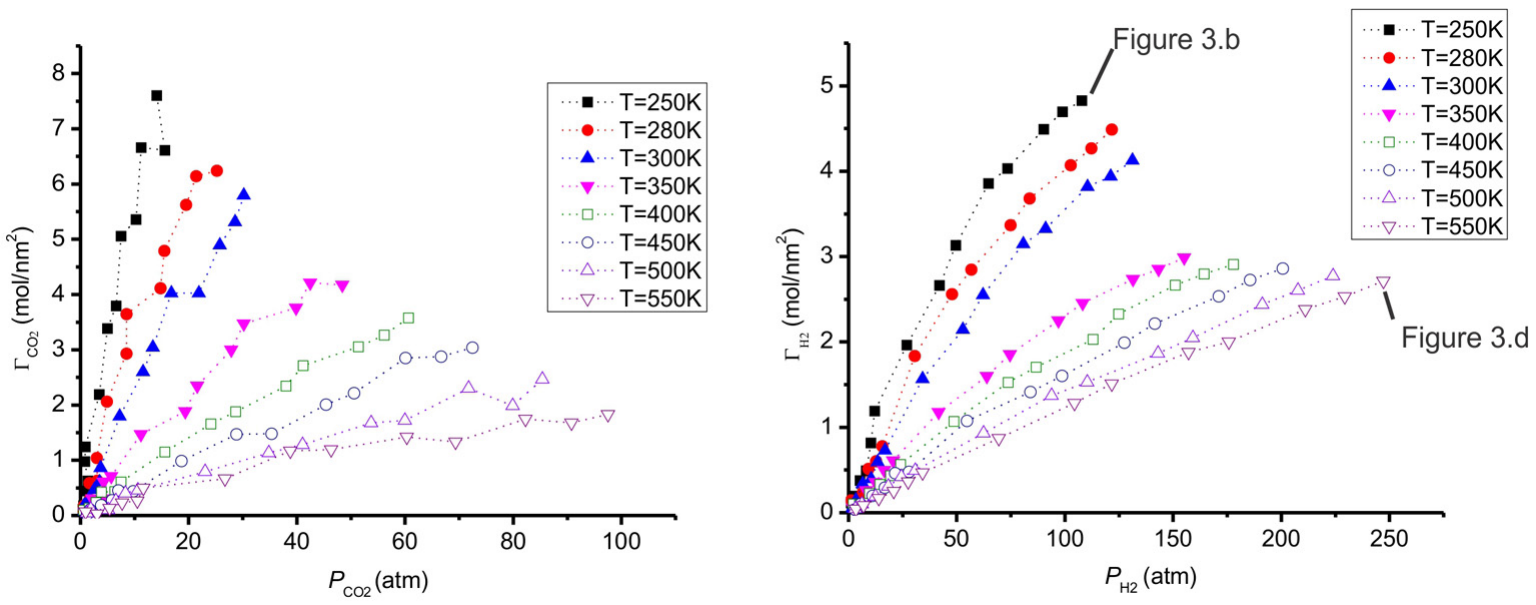
**The adsorption of CO_2_ and H_2_ as a function of partial pressure of CO_2_ (left) and H_2_ (right) at different temperatures**. The states used for the snapshots in Figures 3B,D are shown.

**Figure 7:**
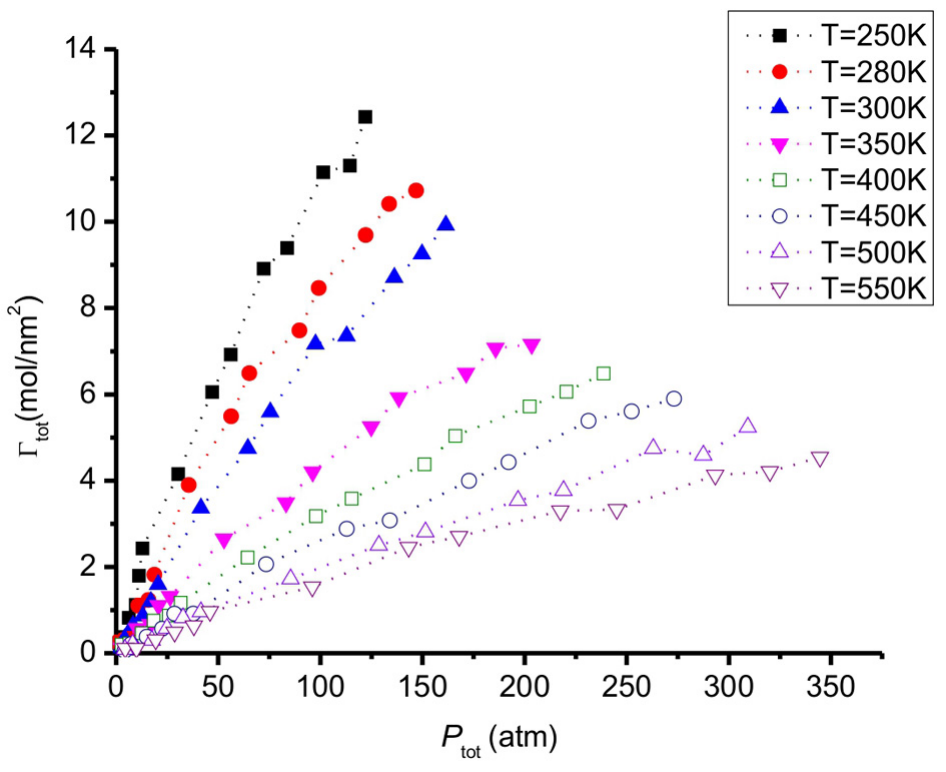
**Total amount of CO_2_/H_2_ adsorbed on a graphite surface as a function of gas pressure at different temperatures**.

The adsorption of CO_2_ and H_2_ at different temperatures are presented in Figure 6. When the temperature increases, the adsorption decreases, as expected. This behavior was also observed with CO_2_/H_2_ mixtures of molar ratios (20:80 and 10:90) on a different carbon pore structure, using Monte Carlo simulations (Vasanth Kumar and Rodríguez-Reinoso, 2012). The adsorption of hydrogen, Γ_H__2_, is much lower than the value of Γ_CO__2_. We explain the preference of CO_2_ to H_2_ on the surface by its stronger interaction with graphite (Table 2). The H_2_ adsorbs less than CO_2_ and prefers the gas phase. The total adsorption of both CO_2_ and H_2_ is shown in Figure 7. When the temperature increases the mixture adsorbs less.

#### Separation of mixtures

The separation ratio *S* (selectivity) of CO_2_/H_2_ mixture is defined as:

(15) $$S=\frac{n{\text{CO}}_{2}(\text{adsorbed})}{n{\text{CO}}_{2}(\text{gas})}\times \frac{n{\text{H}}_{2}(\text{gas})}{n{\text{H}}_{2}(\text{adsorbed})}$$

**Figure 8:**
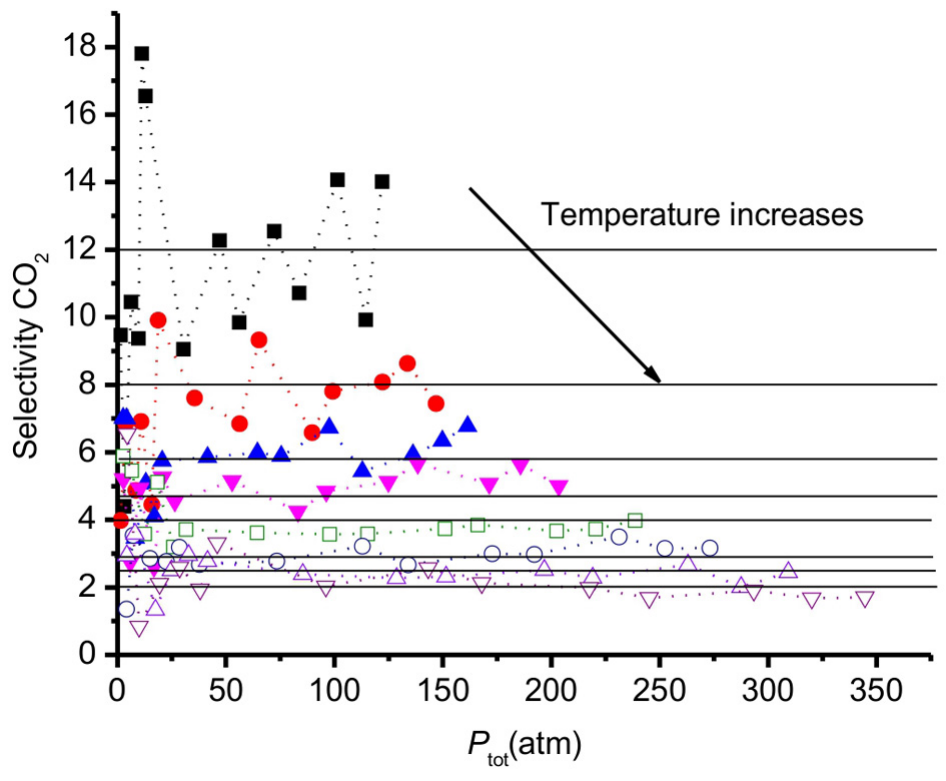
**Selectivity of CO_2_ relative to H_2_*vs*. the total pressure of the system**. Results refer to a graphite surface with pore size 7 nm at different temperatures from 250 to 550 K. The selectivity decreases with increasing temperatures.

The selectivity is commonly used to define the efficiency of a (membrane) material to separate CO_2_ from a mixture of CO_2_, H_2_. For activated carbon, *S* depends on the mixture composition and on the pore sizes (Vasanth Kumar and Rodríguez-Reinoso, 2012). Our surface model can be seen as a graphite crystal with an extra-large slit pore, of 7 nm diameter. The results obtained for *S* are presented in Figure 8. At low pressures (<25 atm), the accuracy in *S* is small (within ±30%) because the small number of molecules leads to poor statistics. At higher pressures (>25 atm), the values of *S* vary around a constant average value within ±10% for each temperature.

Monte Carlo simulations by Kumar et al. (Vasanth Kumar and Rodríguez-Reinoso, 2012) showed that the selectivity increases with increasing pressure with a slit pore geometry. Cao et al. (Cao and Wu, 2005) reported that the selectivity of CO_2_ over H_2_ size decreased when the pressure increased in the low pressure region, using Monte Carlo simulations. The results of Cao et al. can be understood from the selectivity of CO_2_ over H_2_ being maximum for carbon pores around 15 Å (Cao and Wu, 2005), being the double of the surface extension shown in Figure 4. The number of molecules adsorbed larger, relatively speaking, for pore sizes below 2α, leading to high selectivities for such pores or pore distributions. By finding the extension of the surface, one can thus decide on the optimal pore size of the material.

The data in Figure 8 show that the selectivity is essentially invariant of the pressure for pressures above 25 atm. The results indicate that the selectivity goes down below 25 atm. All values *S* are in the range 2–18 and decreases when the temperature increases. At 250K, the average selectivity is 12. At the highest temperature, 550 K, *S* reduces to the average value 2. At high temperatures the two gases have similar adsorption behavior, CO_2_ does not adsorb much stronger than H_2_. This changes at lower temperatures. The trend of *S* is in good agreement with other simulations of CO_2_/H_2_ mixtures in slit pores with smaller pore sizes, using the GCMC technique (Cao and Wu, 2005).

### Surface self-diffusion

The self-diffusion of CO_2_ and H_2_ along the surface was studied. The self-diffusion coefficient of molecule was obtained from:

(16) $$D_{\left|\right|}^{s}=\underset{t\to \infty }{\lim}\left[\frac{1}{2dNt}\sum_{i\;=\;1}^{N}\langle {\left|r_{i}(t)-r_{i}(0)\right|}^{2}\rangle \right]$$

where *d* is the dimensionality (for surface *d* = 2), *N* is the total molecules, *r*_*i*_ (t) and *r*_*i*_ (0) are the projections of the position of molecules on the surface at time *t* and time 0.

All molecules were included in the mean-squared displacement calculations as described in previous studies (Haas et al., 2009). By plotting the logarithm of the diffusion coefficients found vs. the inverse of temperature, we obtained an Arrhenius plot. This was used to estimate the temperature dependence of the diffusion coefficient according to

(17) $$D(T)=D_{0}\exp\left(\frac{-E^{\text{act}}}{RT}\right)$$

where *D*_0_ is the pre-exponential factor, *R* is the gas constant, and *E*act is the activation energy.

**Figure 9:**
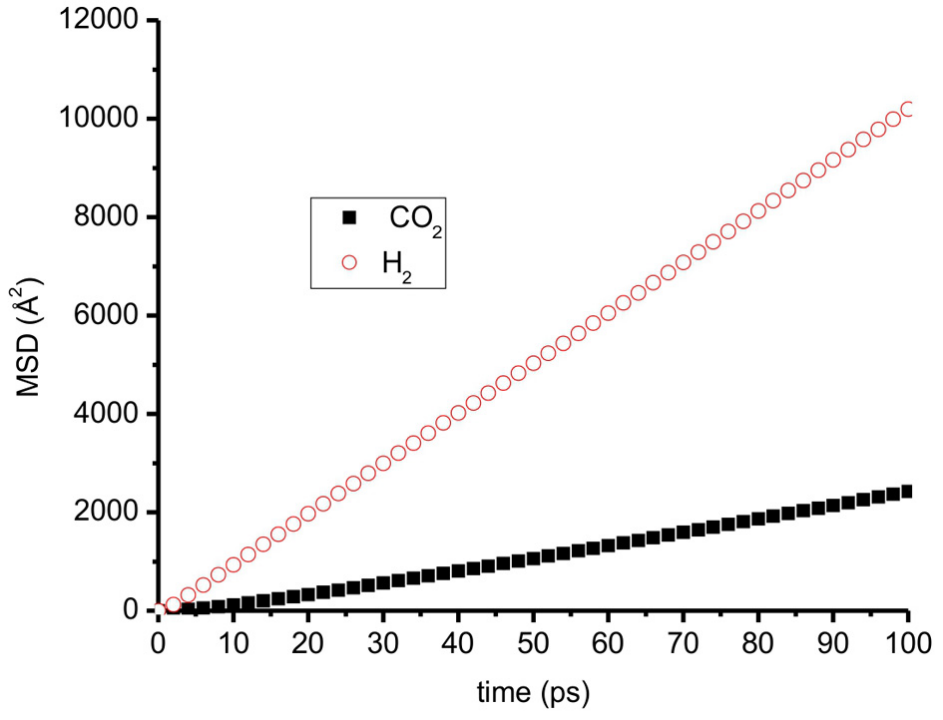
**The mean-squared displacement (MSD) of CO_2_ and H_2_ on a graphite surface at *T* = 300 K, *N* = 700, mole fraction of CO_2_ = 0.3 H_2_ has much higher MSD than CO_2_**.

Figure 9 shows an example of mean-squared displacement of CO_2_ and H_2_ parallel to the graphite surface at temperature 300 K. It is clearly shown that H_2_ diffuses much faster than CO_2_. We observe a similar trend for all cases: H_2_ always has a higher self-diffusion coefficient than CO_2_, because H_2_ is lighter than CO_2_.

**Figure 10:**
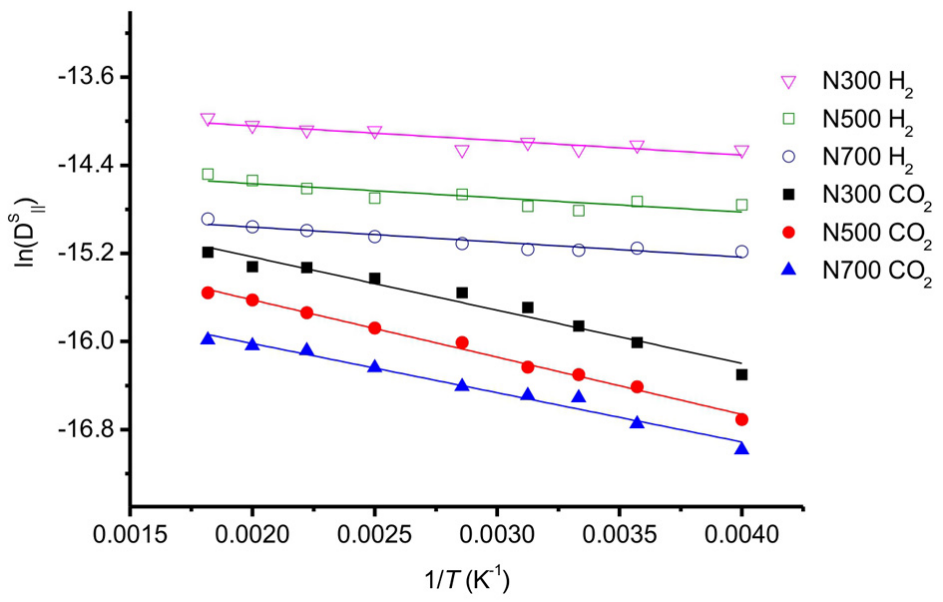
**Natural logarithm of the self-diffusion coefficients as a function of the inverse temperature of selected system**.

The activation barrier for self-diffusion was obtained by calculating the slope of the linear relationship between the natural logarithm of the self-diffusion coefficient and 1/*T* (Figure 10). We found that activation barriers for self-diffusion of CO_2_ varying in the range *E*^act^_CO__2_ = 3.5–4.3 kJ/mol. These values are smaller than the values reported by Lim et al. (2010), giving energy barriers in the range 5.77–6.08 kJ/mol for CO_2_ diffusion. The pores were smaller than 1.0 nm in this case, however. The self-diffusion coefficient of CO_2_ along the graphite surface is higher than values obtained from simulations with smaller pore sizes (<1 nm) (Zhou and Wang, 2000; Lim et al., 2010). This is because larger pores allow surface CO_2_ more space to diffuse, and less interaction between CO_2_ and carbon atoms of graphite. Under the conditions used here with pore size ~7 nm, CO_2_ will diffuse relatively faster and with a smaller diffusion barrier than inside a 1 nm slit pore. This adds to the knowledge on CO_2_ diffusion on graphite surfaces.

For H_2_ on graphite surface, we found self-diffusion barriers in the range *E*^act^_H__2_ = 1.0–1.1 kJ/mol. The self-diffusion barrier for H_2_ is good agreement with experimental data for H_2_ transport on graphite surface. QENS gave 1.0–1.2 kJ/mol, Haas et al. (2009). The *D*^0^_H__2_ in our study was also in a very good agreement with other experimental values (Table 3).

**Table 3:** **Summary of results on self-diffusion parameters cf. Equation (17) for a mixture of CO_2_ and H_2_ on a graphite surface**.

	**This work (simulation) on graphite surface**	**References**
*D*^0^_CO__2_	2.7 × 10^−7^–6.4 × 10^−6^ (m^2^/s)	MD simulation (Zhou and Wang, 2000; Lim et al., 2010) (very small slit pore <1 nm) ~1 × 10^−9^ (m^2^/s)
*E*^act^_CO__2_	3.5–4.3 kJ/mol	(very small slit pore <1 nm) 5.77–6.08 kJ/mol ref (Lim et al., 2010)
*D*^0^_H__2_	4.2 × 10^−7^–1.1 × 10^−6^ (m^2^/s)	QENS experiment (Haas et al., 2009) (graphite surface) 1.9 × 10^−7^ (m^2^/s) for 1 ML 3.5 × 10^−7^ (m^2^/s) for 0.5 ML
*E*^act^_H__2_	1.0–1.2 kJ/mol	QENS experiment (Haas et al., 2009) (graphite surface) 1.0–1.1 (kJ/mol)

**Figure 11:**
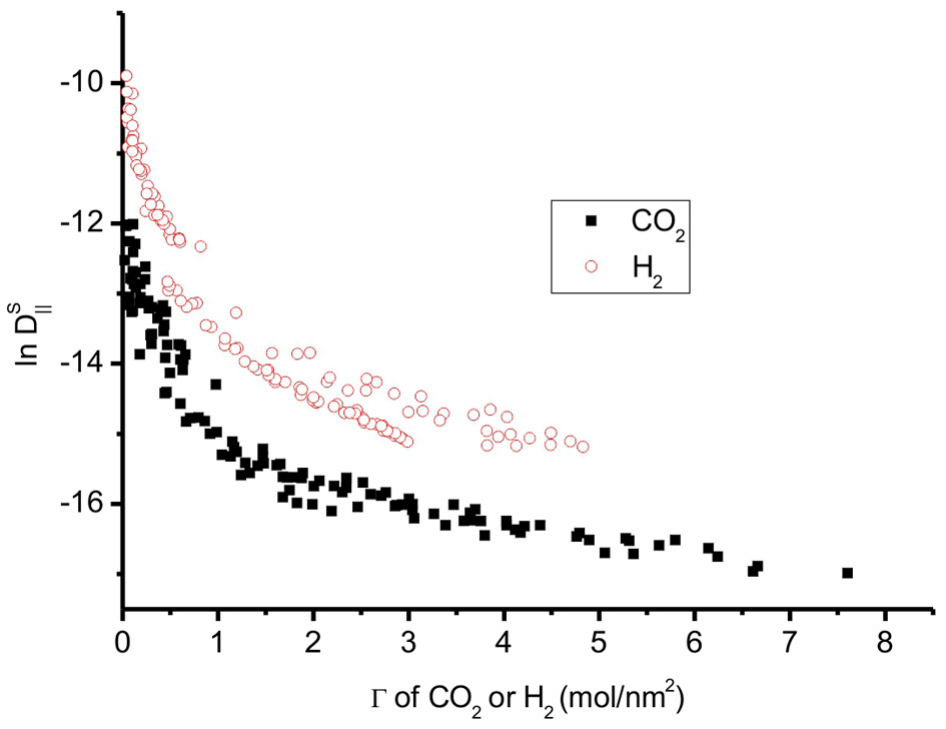
**Natural logarithm of all self-diffusion coefficients *vs*. adsorption of CO_2_ and H_2_**. H_2_ has higher diffusion coefficients than CO_2_.

The diffusion coefficient of H_2_ is larger than that of CO_2_ (Figure 11). The barrier to self-diffusion of CO_2_ is four times larger than that of H_2_ on a graphite surface. The CO_2_ diffusion depends much more on the temperature than that of H_2_. Hence at high temperature, CO_2_, and H_2_ will have similar diffusion coefficients.

The results (Figure 8) have shown that the selectivity is more or less invariant to the total pressure above 25 atm, but highly dependent on the temperature. The permeation of a gas through a membrane is a product of diffusion and adsorption, and the main driving force for separation is given by the partial pressure difference over the membrane. This means that separation of CO_2_ from a mixture with H_2_ at any pressure can best occur at low temperatures, when graphite as an adsorbing material is most effective for CO_2_. The adsorbed CO_2_ will then induce pore size reduction, hence hindering H_2_ to permeate, and hence CO_2_ can be selectively permeated. At high temperature, the permeated gas will be enriched in H_2_ since adsorbed CO_2_ will no longer be blocking the pores, and there will hardly be any selectivity between the two. However, if the pore size can be tailored to the range of 3–4 Å, one may achieve a diffusional selectivity in favor of H_2_ at high temperatures (Figure 10).

The PSA process is based on adsorption at high pressures and desorption at low pressures. By combining PSA with low-high temperature (TSA-process), further enrichment of CO_2_ could be obtained by repeating these equilibrium adsorption steps on activated carbon. By modifying the structure of a graphite surface, one may enhance the separation of CO_2_ out of mixture with H_2_; both when considering a PSA-TSA process as well as for carbon molecular sieve membranes.

## Conclusion

In this work, we have used Equilibrium MD to study the adsorption, selectivity, and self-diffusion of a mixture of CO_2_ and H_2_ (overall mole fraction 0.30 of CO_2_) adsorbed on a slit graphite surface. The results show that there is a preferential adsorption of CO_2_ to H_2_ in the adsorbed layer, which depends on the temperature. CO_2_ adsorbs stronger than H_2_ at low temperatures, while at high temperatures there is little preferential adsorption of CO_2_ over H_2_. The sorption selectivity of CO_2_ over H_2_ on the graphite surface is invariant to pressure above 25 atm, but reduces when temperature increases. The self-diffusion of CO_2_ on graphite surface is the order of magnitude ~10^−8^ m^2^/s. This is larger than for CO_2_ confined in small slit pore by orders of magnitude. The self-diffusion of H_2_ on graphite is in very good agreement with available experimental data (Haas et al., 2009). CO_2_ has a higher energy barrier of diffusion than H_2_. These results of the equilibrium system are useful for process enrichment studies of CO_2_.

### Conflict of interest statement

The authors declare that the research was conducted in the absence of any commercial or financial relationships that could be construed as a potential conflict of interest.
